# Assessing fundamental motor skills proficiency in school-based sports physical education programs: implications for talent development

**DOI:** 10.3389/fspor.2025.1632930

**Published:** 2025-08-21

**Authors:** Hubert Makaruk, E. Kipling Webster, Jared Porter, Beata Makaruk, Anna Bodasińska, Janusz Zieliński, Paweł Tomaszewski, Marta Nogal, Marcin Starzak, Marcin Śliwa, Michał Banaś, Michał Biegajło, Agata Chaliburda, Bogusz Suchecki, Bartosz Molik, Jerzy Sadowski

**Affiliations:** ^1^ Faculty of Physical Education and Health in Biala Podlaska, Józef Piłsudski University of Physical Education in Warsaw, Warsaw, Poland; ^2^ Department of Kinesiology, Recreation, and Sport Studies, University of Tennessee, Knoxville, TN, United States; ^3^ Faculty of Physical Education, Józef Piłsudski University of Physical Education in Warsaw, Warsaw, Poland; ^4^ Faculty of Rehabilitation, Józef Piłsudski University of Physical Education in Warsaw, Warsaw, Poland

**Keywords:** motor competence, motor behavior, physical education, early specialization, youth

## Abstract

**Introduction:**

Fundamental motor skills (FMS) are essential for fostering physical literacy, supporting talent development, and promoting public health in school-aged populations. This study aimed to evaluate FMS proficiency among students in school-based sports physical education (PE) programs, which offer sport-specific training, and compare it to students in traditional PE programs. A secondary aim was to examine whether these programs promote early specialization or early diversification in youth sport development.

**Methods:**

A cross-sectional analysis was conducted on 1,332 students (ages 10–14; 58% boys) from 12 schools across Poland, including 547 students in school-based sports PE programs and 785 in traditional PE. The Fundamental Motor Skills in Sport (FUS) test, a qualitative and process-oriented assessment tool, was used to evaluate FMS across six motor tasks: hurdles, jumping rope, forward roll, ball bouncing, ball throwing and catching, and kicking and stopping a ball. Participants were further categorized by sport: boys into basketball, track and field, soccer, and volleyball; girls into basketball, track and field, and volleyball.

**Results:**

Overall, FMS proficiency was at an “insufficient” level in both groups, with 72% of boys and 77% of girls in school-based sports PE programs, and 90% of boys and 92% of girls in traditional PE programs. Additionally, the analysis revealed a predominant emphasis on early specialization within school-based sports PE programs.

**Conclusions:**

Both school-based sports and traditional PE programs fail to ensure adequate FMS proficiency in students. The sport PE programs' curricular focus on early specialization over diversification may further restrict opportunities for motor competence development, with potential consequences for lifelong physical activity and the early stages of talent development.

## 1 Introduction

In the field of sports sciences, motor competence is commonly defined as an individual's ability to proficiently perform a variety of fundamental movement patterns (1, 2). It represents a foundational construct for motor development, acting as a prerequisite for the acquisition and refinement of more complex and sport-specific skills. These foundational capacities, known as fundamental motor skills (FMS), are essential for successful participation in a wide range of physical activities and serve as the building blocks for long-term engagement in sport and exercise (3). FMS are typically categorized into three domains: locomotor skills (e.g., running, jumping), object control or manipulative skills (e.g., throwing, kicking), and balance or stability skills (e.g., body rolling, static balancing) (3, 4).

A common misconception is that FMS develop spontaneously and without assistance in children; however, evidence shows they necessitate a structured and sometimes challenging learning process, requiring guidance and reinforcement from physical education (PE) teachers, sports coaches, and parents (5, 6). Failure to acquire these skills during key developmental windows can limit participation and performance later in life. Conversely, early mastery of FMS sets a positive developmental trajectory that increases the likelihood of sustained physical activity (PA), enhanced fitness, and improved psychosocial well-being throughout adolescence and adulthood (7, 8). This concern becomes more pressing given global data showing that over 80% of adolescents aged 11–17 fail to meet recommended daily levels of PA (9). Such widespread inactivity is linked to a range of long-term health risks, including childhood obesity (10), cardiometabolic disorders (11), and mental health challenges such as anxiety and depression (12). Therefore, early and structured development of motor competence through FMS engagement is increasingly prioritized as a key strategy in public health initiatives targeting school-aged populations (13).

Research in the field of sport sciences indicates that children with higher FMS proficiency not only perform better in complex, sport-specific tasks but also adapt more effectively to the technical, tactical, and physical demands of performance (14, 15). These children not only learn new tasks more efficiently but also exhibit greater skill transferability across different sports and activities. In contrast, children with low FMS tend to experience the so-called “proficiency barrier”, a developmental stage after which skill acquisition becomes increasingly complex (16). This barrier may hinder their ability to fully engage in more advanced forms of movement, limiting both participation and progression within organized sport. The existence of this barrier emphasizes the necessity for early exposure to developmentally appropriate, varied, and intrinsically motivating movement experiences. It also highlights the need for youth sport systems to adopt approaches that prioritize motor skill development before emphasizing intensive specialization.

In this context, two primary models of athlete development are commonly discussed: early specialization and early diversification (17). Early specialization involves participation in a single sport from a young age, combined with structured, high-volume training commonly referred to as deliberate practice (18), which is intended to maximize performance outcomes. This approach is common in sports with early peak age requirements, such as artistic gymnastics and figure skating (19, 20). However, despite its prevalence, recent research has questioned its long-term efficacy and raised concerns regarding associated risks, including overuse injuries, burnout, and reduced enjoyment (21, 22). By contrast, the early diversification model, as described in the Developmental Model of Sport Participation (DMSP), promotes engagement in a variety of sports during childhood through deliberate play—activities that are self-motivated, loosely organized, and socially enjoyable (23, 24). This model encourages a delayed approach to specialization, typically beginning in adolescence, and comprehensive skill development. Importantly, it also supports FMS development across domains, thereby helping children overcome the proficiency barrier (16, 17). Longitudinal findings suggest that many elite athletes specialized later and engaged in a wider range of sports than their peers (25, 26). This pattern is further supported by a recent meta-analytic review, which found that adult world-class athletes generally began intensive training later and accumulated more multi-sport experience in youth than their national-level counterparts (27). Around the ages of 12–15, defined as the specialization years, adolescents begin to narrow their sport involvement and gradually increase structured training while still maintaining elements of enjoyment and exploration.

Although models such as the DMSP offer clear conceptual guidance (23), a considerable gap may still exist between their theoretical foundations and their implementation in real-world settings. For example, sport-specialized schools (28), where PE is integrated with intensive training in a single chosen sport, are often viewed as having the potential to support both athletic performance and health-related fitness. Instruction in these settings is typically delivered by qualified coaches in collaboration with PE teachers. In addition to extended training time and access to professional coaching, these programs often provide structured daily routines, improved access to sport-specific facilities, and opportunities for peer bonding within sport groups. These elements may, in turn, contribute positively to students' motivation, discipline, and psychological well-being. However, the design and objectives of such programs vary across countries. In many European nations, sport-specialized schools are embedded within the formal education system, integrating intensive sport training into the school curriculum alongside academic subjects to support early talent development (29). By contrast, in Scandinavian countries, youth sport development is primarily organized through club-based systems that emphasize voluntary participation and broader motor skill development during early adolescence.

In practice, the structure and pedagogical emphasis of sport-specialized schools may increasingly reflect the goals of high-performance sport rather than the broader developmental objectives of PE. As a result, these programs often focus on refining sport-specific skills under competitive demands and the expectations of sport federations. This focus can reduce opportunities for varied motor experiences, gradual skill progression, and alignment with individual readiness (30). Under such conditions, the holistic development of overall FMS proficiency may be compromised or deprioritized, despite its central role in PE curricula. To date, relatively little research has explored whether these environments effectively foster a broad base of motor skills or predominantly reinforce narrow competencies in youth already oriented toward competitive sport (17, 27). This uncertainty raises important questions about access, program effectiveness, and long-term developmental outcomes. These concerns are relevant not only for young athletes but also for the wider student population. It is therefore essential to critically assess how sport-focused school programs function in real-world settings: do they foster FMS development among all students, or do they primarily serve those already progressing along a pathway of early specialization?.

Previous research has largely focused on elite training systems or general PE, often overlooking hybrid environments such as sport-specialized schools, where educational and athletic objectives converge. In light of this, early specialization is typically identified through indicators such as high training volume, limited sport diversity, and early withdrawal from alternative physical activities or sports (17, 21). To move beyond retrospective evaluation of long-term outcomes, there is a growing need for early-stage markers that reflect the developmental orientation of such programs. In this regard, FMS proficiency represents a theoretically grounded and practically applicable indicator capable of signaling whether a training environment aligns more closely with early diversification or early specialization models. FMS profiling offers a practical method for assessing whether training environments promote broad-based skill development or reinforce early specialization (21, 30). This reinforces the need for early and actionable indicators to distinguish between contrasting developmental pathways. When measured using standardized and domain-specific assessments, FMS proficiency serves as a valid yet underused metric for evaluating whether a program cultivates comprehensive motor competence or channels athletes into narrow performance trajectories (17).

Accordingly, the present study had two principal aims: (i) to assess the overall FMS proficiency of students attending school-based sports PE programs compared to those in traditional PE settings, and (ii) to examine whether distinct sport-specific tracks within these programs exhibit characteristics aligned with early specialization or early diversification. These findings are expected to inform the optimization of athlete development pathways by supporting both competitive sport progression and the promotion of lifelong engagement in PA.

## 2 Methods

### 2.1 Participants

Cross-sectional data were gathered for this study within the framework of a nationwide program aimed at promoting and assessing physical fitness, PA and FMS among school-aged students, known as “Physical Education (WF) with University of Physical Education (AWF)—Active today for a healthy future.” Twelve schools, representing 12 out of 16 provinces across Poland, were randomly selected from schools participating in the program (*n* = 192) and were stratified based on their location (rural, town, or city) and level of sports involvement (school-based sports PE program or school-based traditional PE program). The consent rate for participation in the study was 96%. The sample consisted of 1,332 children and adolescents in grades 4–8 (aged 10–14 years), with 774 boys and 558 girls. Additionally, 547 students participated in a school-based sports PE program (with at least 10 h of PE classes per week), while 785 students were enrolled in a traditional school program (with 4 h per week). For further analysis, participants were divided into four sport categories for boys: basketball (*n* = 72), track and field (*n* = 102), soccer (*n* = 65), and volleyball (*n* = 93), and three categories for girls: basketball (*n* = 38), track and field (*n* = 71), and volleyball (*n* = 106). Written parental consent was obtained prior to data collection, and the study design and testing protocol were approved by an institutional Research Ethics Committee under protocol number SKE 01-19/2022.

### 2.2 Measurements and procedures

The current investigation employed the FUS test (Test of Fundamental Motor Skills in Sport), a qualitative and process-oriented assessment tool designed to evaluate FMS proficiency (32). The test comprises six movement skills: hurdles, jumping rope, forward roll, ball bouncing, ball throwing and catching, and kicking and stopping a ball. Each skill includes five criterion-referenced behavioral components, which were evaluated to determine the level of skill mastery. The FUS test incorporates developmental adjustments designed to provide age-appropriate levels of difficulty while ensuring that the scoring criteria remain identical and directly comparable across the entire 10–14 age range. For example, in the hurdles task, hurdle heights were set at 50 cm for students aged 10–12 and 60 cm for those aged 13–14. In the throwing and catching task, students aged 13–14 were required to catch the ball with one hand after it rebounded from the wall and performed the task from a distance of 6 m, whereas students aged 10–12 caught the ball with both hands from 5 m. Similarly, in the kicking and stopping task, students aged 13–14 kicked the ball toward a slightly smaller target area on the wall (3  ×  2.5 m) from a distance of 6 m, while students aged 10–12 aimed at a larger target area (3 × 3 m) from 5 m. For the remaining tasks, jumping rope, forward roll, and ball bouncing, the same conditions were applied across all ages.

Scoring for each criterion of the test is binary, with a score of “1” indicating the criterion was met, and “0” denoting it was not. Two attempts were provided for each item, and the trial yielding the higher score was utilized for subsequent analysis. Mastery levels were defined for each skill, delineated as follows: “full mastery”, denoting all components executed correctly and scoring 5 points; “near mastery”, indicating all but one component performed correctly, earning 4 points; 'some mastery', reflecting three components performed correctly, yielding 3 points; and “poor”, signifying two or fewer components executed correctly. Overall FMS proficiency was evaluated across all six FUS skills at four levels. “Excellent FMS proficiency” was attained if the student fully mastered all six assessed skills or mastered all but one at the “near mastery” level. “Good FMS proficiency” was achieved if the student achieved at least “near mastery” for each FUS skill and did not meet the criteria for “excellent FMS proficiency”. “Elementary FMS proficiency” was reached if the student scored at least “some mastery” level for each skill and did not meet the requirements for “excellent” or “good” proficiency. “Insufficient FMS proficiency” was assigned if the student did not meet the criteria for any of the other proficiency levels.

The FUS test has previously demonstrated excellent psychometric properties, including high content validity (CVI ≥ 0.83), substantial to almost perfect inter-rater reliability (Cohen's kappa = 0.75–0.86, ICC = 0.95–0.98), and excellent intra-rater and test-retest reliability (ICC = 0.95–0.97), supporting its suitability for assessing FMS in school-aged populations (32, 33).

### 2.3 Data collection and analysis

All FUS assessments followed the standardized protocol published in Makaruk et al. (32) and were conducted between April and June 2023 during scheduled PE classes. Testing was carried out by six 4-person research teams composed of qualified PE teachers, movement science researchers, and doctoral students in sport and health sciences, all of whom completed formal training on the FUS testing manual and scoring criteria (32). Each assessor was familiar with the tool's structure, motor criteria, and video analysis procedures. After a dynamic warm-up, students were divided into four groups, each assigned to one of four testing stations (1. hurdles, 2. jumping rope and forward roll, 3. ball bouncing, 4. throwing and catching, as well as kicking and stopping a ball). Before testing each skill, participants received a brief explanation and demonstration of how to perform the task and which components would be evaluated. Students completed one familiarization trial for each task, followed by two testing trials without any augmented feedback. Each testing session accommodated between 12 and 24 students and lasted approximately 45–50 minutes, conducted both indoors and outdoors. Each trial was recorded using a tripod-mounted video camera (Lamax W 9.1, Poland) in MP4 format, with a resolution of 1,920 × 1,080 pixels. Specific recording methods, distances, and camera angles were predetermined for each task. Each skill was scored by a pair of raters using retrospective video analysis. Raters independently evaluated the recordings, and any discrepancies in scoring were resolved through discussion or third-party review. For a comprehensive guide to the FUS testing procedure, please refer to the “Test of Fundamental Motor Skills in Sport” manual for teachers (34).

### 2.4 Statistical analysis

Basic descriptive statistics, such as means, standard deviations, and percentages, were used to present the results. Differences in contingency tables for levels of movement skills were assessed using the chi-square (*χ*²) test separately for sex (boys/girls), type of PE program (regular/sport), and school-based sport program specialization (basketball, track and field, soccer, volleyball). Cramer's V was used as a measure of effect size with cutoffs of 0.1, 0.3, and 0.5 for small, medium, and large effects, respectively (35). Due to significant deviations from the assumptions of parametric tests, non-parametric procedures were used for comparing quantitative data. Mean scores of individual FMS tasks between boys and girls, and between traditional and sport PE programs, were compared using the Mann–Whitney test; the corresponding Z-score was reported, and additionally, the r-equivalent measure (req) was calculated to express the effect size, as recommended for nonparametric procedures (36). The Kruskal–Wallis test was used to compare the performance of boys and girls in individual FMS tasks across different school-based sports PE program specializations. Sports-related differences in the percentages of participants achieving mastery and near mastery in individual FMS tasks were assessed using the two-sample *Z*-test for proportions, with Bonferroni correction for multiple comparisons, i.e., between school-based sports PE programs. The level of statistical significance was set at alpha = .05. Data were analyzed using SPSS 27 for Windows (SPSS Inc., Chicago, USA).

## 3 Results

Table 1 presents the distribution of overall FMS proficiency levels by sex and PE program type. In the school-based sports PE group, the majority of students demonstrated “insufficient FMS proficiency” (boys: 71.7%; girls: 77.2%), yet these rates were significantly lower than those observed in traditional PE (boys: 89.6%; girls: 91.8%; *χ*² = 41.0 for boys and 23.8 for girls, both *p* < 0.001). The corresponding effect sizes (Cramer's V = 0.23 for boys, 0.17 for girls) indicate small associations between program type and proficiency levels, showing clear distributional differences despite some overlap. Although only a small proportion of students in sport PE programs achieved an “excellent” level of FMS proficiency, these programs were associated with a noticeably greater representation in the “good” category (boys: 12.6%; girls: 5.6%; *χ*² = 25.4, *p* < 0.001, V = 0.18 for boys; *χ*² = 9.47, *p* < 0.01, V = 0.13 for girls) and in the “elementary” category (boys: 14.5%; girls: 16.7%; *χ*² = 11.5, *p* < 0.001, V = 0.12 for boys; *χ*² = 14.1, *p* < 0.001, V = 0.16 for girls) compared to their peers in traditional PE.

**Table 1:** Percentage of primary school students at each level of overall FMS proficiency by sex and type of PE program (*n* = 1,332).

Level of overall FMS proficiency	Boys		Girls	
	Sport PE program	Traditional PE program	Sport PE program	Traditional PE program
	(*n* = 332)	(*n* = 442)	(*n* = 215)	(*n* = 343)
Excellent FMS proficiency	1.20	0.23	0.47	0.58
Good FMS proficiency	12.65	3.17*	5.58**	0.87*
Elementary FMS proficiency	14.46	7.01*	16.74	6.71*
Insufficient FMS proficiency	71.69	89.59*	77.21	91.84*

* Significantly (*p* < .05) different from sport PE program.

** Significantly (*p* < .05) different from boys involved sport PE program.

Table 2 shows group-level differences in performance on individual FMS tasks. Boys and girls enrolled in the sport PE programs achieved significantly higher average scores than their peers in the traditional PE programs across all six assessed tasks. The largest differences were found in forward roll (boys: Z = 10.15, *p* < .001, req = 0.371; girls: Z = 7.12, *p* < .001, req = 0.301), while the smallest were in kicking and stopping a ball (boys: Z = 3.96, *p* < .001, req = 0.150; girls: Z = 1.90, *p* = .057, req = 0.080). Accordingly, students from sport PE programs were more likely to reach “full mastery” or “near mastery” levels across individual tasks, with differences more pronounced in boys (13.3%–31.3%) than in girls (3.6%–25.5%). No significant age differences were observed between students across PE program types (sports PE = 11.8 ± 1.21; traditional PE = 11.7 ± 1.25).

**Table 2:** Mean ± SD score and percentage of participants achieving mastery and near mastery in individual FMS within the FUS test tasks among school students, categorized by sex and specialization in PE program.

FMS task	Boys		Girls	
	Sport PE program	Traditional PE program	Sport PE program	Traditional PE program
	(*n* = 332)	(*n* = 442)	(*n* = 215)	(*n* = 343)
Hurdles	3.08 ± 1.64	2.05 ± 1.71*	2.80 ± 1.59**	1.94 ± 1.63*
	50.9%	26.5%	40.9%	23.0%
Jumping rope	2.27 ± 1.98	1.46 ± 1.87*	3.28 ± 1.72**	2.42 ± 1.93*^,^**
	36.8%	20.6%	57.7%	36.4%
Forward roll	3.47 ± 1.50	2.17 ± 1.70*	3.81 ± 1.40**	2.74 ± 1.73*^,^**
	59.6%	28.3%	55.8%	39.1%
Ball bouncing	3.58 ± 0.95	2.82 ± 1.15*	3.17 ± 0.87**	2.48 ± 1.11*^,^**
	56.3%	29.2%	35.8%	15.7%
Throwing and catching	3.45 ± 0.93	2.92 ± 1.11*	3.14 ± 1.02**	2.32 ± 1.17*^,^**
	58.1%	34.2%	40.5%	15.4%
Kicking and stopping a ball	4.03 ± 1.31	3.77 ± 1.20*	2.67 ± 1.30**	2.47 ± 1.34**
	74.4%	61.1%	26.1%	22.5%

* Significantly (*p* < .05) different from sport PE program.

** Significantly (*p* < .05) different from boys.

To explore potential links between sport-specific school tracks and FMS outcomes, students in the school-based sports PE programs were further analyzed by their sport focus (basketball, soccer, track and field, volleyball). Table 3 presents overall FMS proficiency levels among boys across these program types. The highest percentage of boys (20.6%) classified at the “elementary FMS proficiency” level was observed in track and field, which significantly outperformed volleyball (*χ*² = 4.45, *p* = .035, Cramer's V = 0.15). In contrast, volleyball programs showed the highest rate (79.6%) of “insufficient FMS proficiency”, particularly compared to track and field (*χ*² = 6.66, *p* = .01, Cramer's V = 0.18). These effect sizes (Cramer's V = 0.15–0.18) indicate small associations between sport tracks and FMS proficiency. Consequently, the differences in FMS levels between sports tracks have limited practical importance. Notably, no boys from the basketball or volleyball groups reached the “excellent FMS proficiency” level. Age differences between groups were not significant (mean ± SD: basketball = 11.8 ± 1.5, track and field = 11.9 ± 1.6, soccer = 11.6 ± 1.4, volleyball = 11.7 ± 1.4).

**Table 3:** Percentage of boys at each level of overall FMS proficiency by school-based sports PE program specialization (*n* = 332).

Level of overall FMS proficiency	Basketball	Track and Field	Soccer	Volleyball
	(*n* = 72)	(*n* = 102)	(*n* = 65)	(*n* = 93)
Excellent FMS proficiency	1.39	2.94	0	0
Good FMS proficiency	9.72	13.73	16.92	10.75
Elementary FMS proficiency	12.50	20.59	13.85	9.68*
Insufficient FMS proficiency	76.39	62.75	69.23	79.57*

* Significantly (*p* < .05) different from track and field.

Table 4 illustrates differences in overall FMS proficiency among girls enrolled in various sport-focused PE tracks (basketball, track and field, volleyball). Girls in track and field performed better overall, with significantly fewer classified in the “insufficient” category (66.2%) compared to girls in basketball (86.8%, *χ*² = 5.40, *p* = .020, Cramer's V = 0.22) and volleyball (81.1%, *χ*² = 5.08, *p* = .024, Cramer's V = 0.17). The effect sizes (Cramer's V = 0.17–0.22) indicate small associations between sport specialization type and FMS proficiency among girls. Again, no significant age differences were found across groups (basketball = 11.8 ± 1.6, track and field = 11.8 ± 1.6, volleyball = 11.4 ± 1.2).

**Table 4:** Percentage of girls at each level of overall FMS proficiency by school-based sports PE program specialization (*n* = 215).

Level of overall FMS proficiency	Basketball	Track and Field	Volleyball
	(*n* = 38)	(*n* = 71)	(*n* = 106)
Excellent FMS proficiency	0	1.41	0
Good FMS proficiency	2.63	8.45	4.72
Elementary FMS proficiency	10.53	23.94	14.15
Insufficient FMS proficiency	86.84*	66.20	81.13*

* Significantly (*p* < .05) different from track and field.

The performance of boys in individual FMS tasks across different school-based sports PE program specializations is presented in Table 5. Soccer participants achieved the highest mean score in kicking and stopping a ball (4.82 ± 0.58), significantly outperforming participants in other programs (H = 42.33, *p* < .001). Basketball boys demonstrated superior mean proficiency in ball bouncing (4.29 ± 0.66), with scores significantly higher than those of participants in track and field, soccer, and volleyball (H = 79.09, *p* < .001). Track and field students achieved high mean scores in forward roll (4.07 ± 1.18) and hurdles (3.83 ± 1.36), reflecting their relative strength in these tasks (H = 38.88, *p* < .001 for forward roll; H = 47.71, *p* < .001 for hurdles). For jumping rope, a significant overall difference was observed between programs (H = 8.02, *p* < .05). No significant differences were found for throwing and catching (H = 2.06, *p* = .56).

**Table 5:** Mean ± SD score of individual FMS in the FUS test tasks among boys (*n* = 332) by school-based sports PE program specialization.

FMS task	Basketball	Track and Field	Soccer	Volleyball
	(*n* = 72)	(*n* = 102)	(*n* = 65)	(*n* = 93)
Hurdles	2.29 ± 1.53	3.83 ± 1.36*	2.62 ± 1.48**	3.19 ± 1.75*
Jumping rope	2.81 ± 1.89	2.07 ± 2.01	1.89 ± 1.94	2.33 ± 1.97
Forward roll	2.68 ± 1.73	4.07 ± 1.18*	3.75 ± 1.33*	3.24 ± 1.41**
Ball bouncing	4.29 ± 0.66	3.15 ± 0.87*	3.88 ± 0.78*^,^**	3.31 ± 0.94*^,^***
Throwing and catching	3.35 ± 0.92	3.42 ± 0.98	3.57 ± 0.83	3.47 ± 0.96
Kicking and stopping a ball	3.50 ± 1.92	4.07 ± 0.89	4.82 ± 0.58*^,^**	3.85 ± 1.22***

* Significantly (*p* < .05) different from basketball.

** Significantly (*p* < .05) different from track and field.

*** Significantly (*p* < .05) different from soccer.

**Figure 1:**
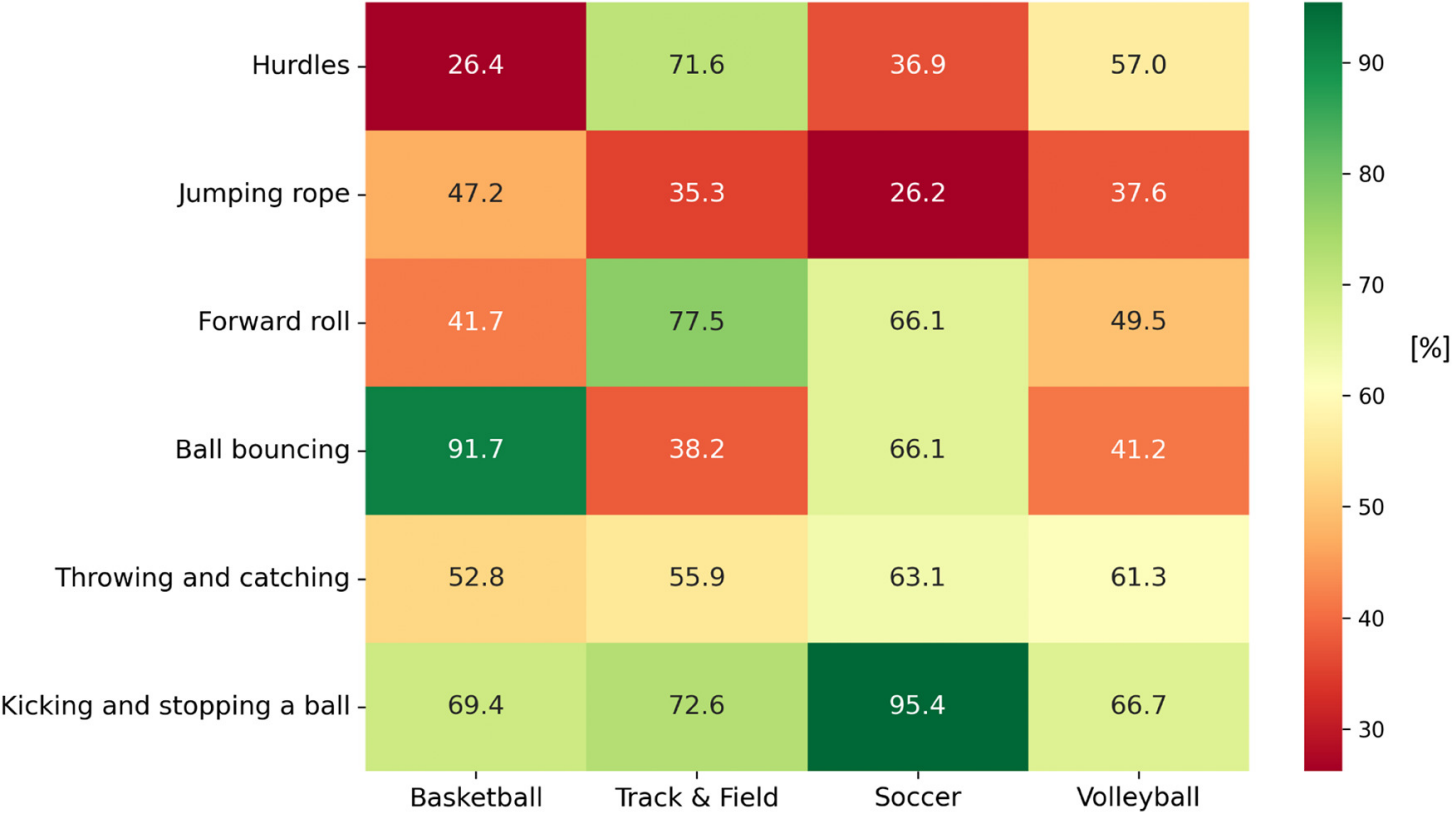
Percentage of participants achieving “mastery” and “near mastery” in individual FMS within the FUS test tasks among boys (*n* = 332) by school-based sports PE program specialization.

Figure 1 visually depicts the percentage of boys achieving “mastery” or “near mastery” for each FMS task by sport program. Soccer participants excelled in kicking and stopping a ball (95.4%), while basketball boys reached the highest proficiency in ball bouncing (91.7%). Track and field boys also performed strongly in forward roll (77.5%) and hurdles (71.6%).

The FMS performance of girls across school-based sports PE programs is shown in Table 6. Track and field participants achieved the highest mean scores in hurdles (3.70 ± 1.59) and forward roll (4.23 ± 1.14), significantly outperforming girls in basketball and volleyball (H = 57.04, *p* < .001 for hurdles; H = 9.79, *p* < .01 for forward roll). In contrast, basketball girls excelled in ball bouncing (3.87 ± 0.81) and jumping rope (3.47 ± 1.72), achieving significantly higher mean scores than participants in track and field and volleyball (H = 30.59, *p* < .001 for ball bouncing; H = 15.77, *p* < .001 for jumping rope). Volleyball participants performed best in throwing and catching (3.36 ± 1.08), significantly outperforming girls in track and field and basketball (H = 14.43, *p* < .001). For the kicking and stopping a ball task, track and field girls again achieved the highest mean scores (3.39 ± 0.87), with volleyball participants scoring lowest (H = 34.72, *p* < .001).

**Table 6:** Mean ± SD score of individual FMS in the FUS test tasks among girls (*n* = 215) by school-based sports PE program specialization.

Task	Basketball	Track and Field	Volleyball
	(*n* = 38)	(*n* = 71)	(*n* = 106)
Hurdles	1.42 ± 1.00	3.70 ± 1.59*	2.68 ± 1.36*^,^**
Jumping rope	3.47 ± 1.72	2.66 ± 1.73*	3.63 ± 1.60**
Forward roll	3.47 ± 1.66	4.23 ± 1.14*	3.66 ± 1.41**
Ball bouncing	3.87 ± 0.81	2.96 ± 0.85*	3.07 ± 0.78*
Throwing and catching	2.66 ± 1.05	3.08 ± 0.82	3.36 ± 1.08*
Kicking and stopping a ball	2.53 ± 1.43	3.39 ± 0.87*	2.24 ± 1.30**

* Significantly (*p* < .05) different from basketball.

** Significantly (*p* < .05) different from track and field.

**Figure 2:**
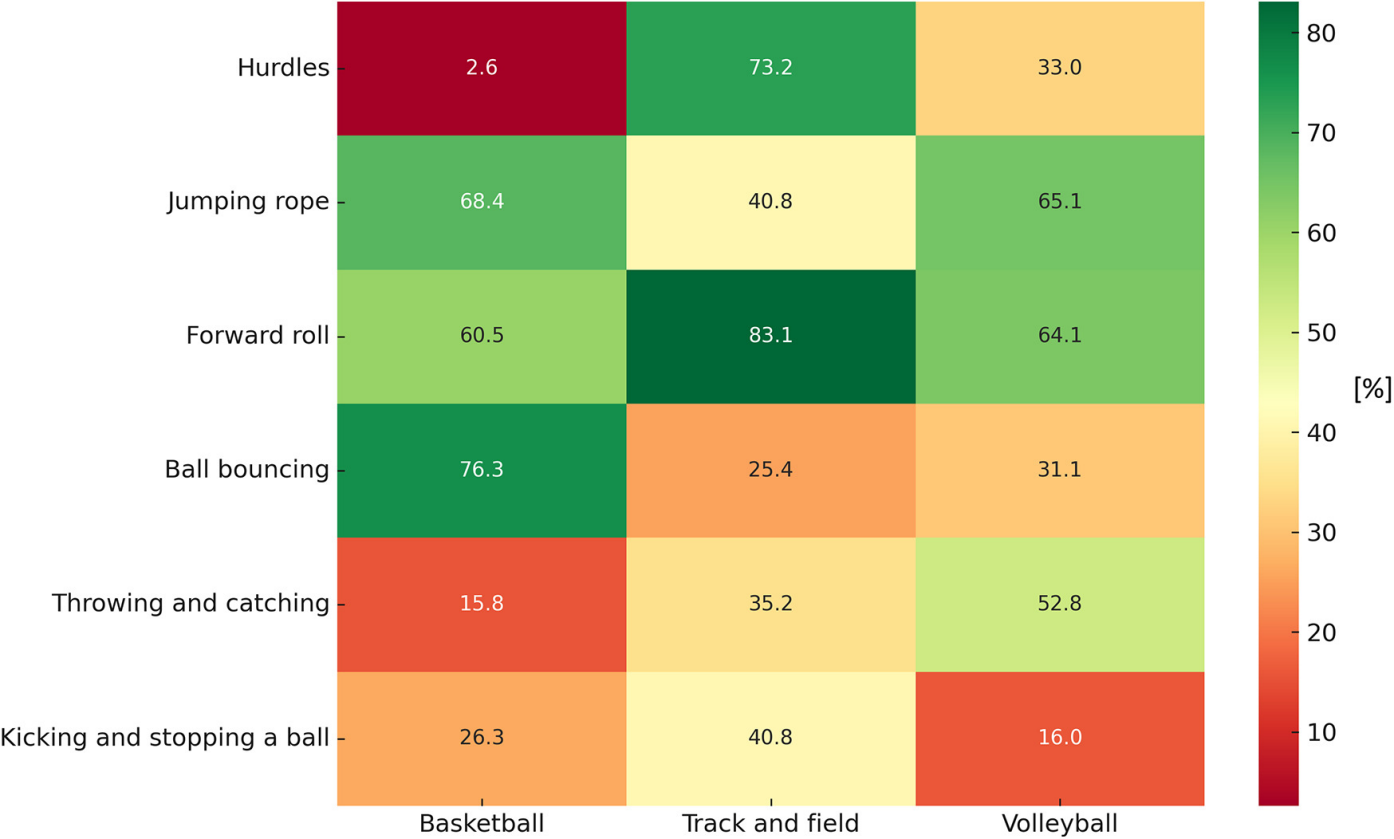
Percentage of participants achieving “mastery” and “near mastery” in individual FMS within the FUS test tasks among girls (*n* = 215) by school-based sports PE program specialization.

Figure 2 illustrates the percentage of girls achieving “mastery” or “near mastery” across tasks. Track and field participants showed leading percentages in hurdles (73.2%) and forward roll (83.1%). Conversely, basketball girls demonstrated the highest proficiency in ball bouncing (76.3%) and jumping rope (68.4%). Volleyball participants excelled in throwing and catching (52.8%), outperforming girls in track and field and basketball.

## 4 Discussion

This study assessed the FMS proficiency of students in school-based sports PE programs and examined whether these programs align more closely with early specialization or diversification models. The findings highlight a critical concern: although students in sport-focused PE programs performed significantly better than their peers in traditional PE, the overall proficiency in these programs remained alarmingly low. Approximately 72% of boys and 77% of girls in sport-specialized PE programs failed to reach even an “elementary” level of FMS proficiency, highlighting substantial gaps in motor competence development despite their participation in structured, sport-specific training. Furthermore, the data revealed that these programs predominantly emphasize sport-specific skills at the expense of overall FMS proficiency, a structural characteristic consistent with early specialization models.

The observation that only 14% of boys and 6% of girls in sport-specialized PE programs achieved a “good” or “excellent” level of FMS proficiency calls into question the developmental adequacy of these programs as early talent pathways. Recent evidence has underscored similar concerns. Butler et al. (37) demonstrated that even highly active middle-school athletes frequently fail to achieve motor skill proficiency across gross motor domains, regardless of their level of sports specialization. Their findings suggest that participation in structured sport alone is insufficient for developing FMS proficiency. Limitations in FMS are particularly concerning in the context of sampling-based talent development models, such as the DMSP (23, 24). Although early specialization models may lead to short-term improvements in specific sport-related skills, these gains often come at the cost of overall FMS proficiency, which forms the foundation for long-term talent development. Without this foundation, athletes may struggle to transition between sports, adapt to increasing training demands, and prevent injuries over time (17, 31). Our study revealed narrow FMS profiles focused on discipline-specific skills. For example, students involved in basketball showed strong performance in ball bouncing, and those focused on soccer excelled in kicking and stopping the ball; however, both groups underperformed in tasks outside their primary sport. These outcomes reflect the influence of sport-specific training environments, where repeated practice in a limited set of tasks may support isolated skill development but not overall FMS proficiency. This one-sided progression may lead to what is known as the “proficiency barrier,” a threshold beyond which further improvement becomes limited if adequate foundations are not established earlier (38, 39). The consequences of this barrier extend beyond slowed technical growth and may also impair students' self-efficacy, enjoyment, and long-term persistence, key attributes for long-term athlete development (23–25).

The finding that 72% of boys and 77% of girls in school-based sports PE programs achieved only “insufficient” FMS proficiency is particularly concerning when viewed through the lens of long-term PA engagement. According to the conceptual model proposed by Stodden et al. (7), motor competence plays a foundational role in promoting sustained PA, particularly during the transition from childhood to adolescence. Students with inadequate proficiency in FMS may not only encounter challenges in sport performance but also develop lower perceived competence, reduced enjoyment, and diminished motivation to remain active outside structured settings (7, 8). The observed deficits in this study suggest that many participants, despite their heightened exposure to organized sport, may lack the essential movement foundations needed to remain physically active as they age – raising concerns about whether current sport-specialized curricula effectively prepare students for both early competition and lifelong PA engagement (17). This underscores the need to reframe youth sport education as a vehicle for promoting inclusive physical literacy, not merely as a pipeline for early athletic performance.

Beyond individual performance patterns, it is also critical to examine the educational systems and instructional contexts shaping these outcomes, particularly within the broader framework of how PE is delivered (28, 29). In many school programs, PE curricula do not include clear and consistent guidelines for developing and assessing FMS, which are often given less emphasis than sport performance goals, participation numbers, or general fitness outcomes. This lack of clarity contributes to variation in teaching approaches and educational priorities between schools. The problem is further complicated by gaps in many PE teacher education programs, where future teachers may receive limited preparation in how to assess in PA (40). By contrast, other countries invest in comprehensive teacher training programs that equip educators with modern pedagogical tools and approaches to support diverse and adaptive teaching methods (41). In more performance-oriented systems school sport often mirrors elite training environments, narrowing instructional focus and limiting opportunities for motor competence development (29, 42). These international examples underscore the importance of designing PE systems that not only prepare athletes for competitive pathways but also support the foundational motor skills necessary for all students, regardless of athletic aspirations (42). Addressing these challenges requires improvements in both initial teacher education and ongoing professional development, alongside updates to local and national PE curricula. Giving FMS a stronger place in school-based PE would help bring teaching practice more in line with current understandings of motor learning and long-term sport participation goals.

Despite participating in the same structured, sport-specialized PE programs, noticeable sex differences in FMS performance were observed. Boys generally performed better in object control skills such as kicking, throwing, and ball bouncing. This pattern may reflect a combination of biological predispositions (43) and long-standing cultural influences (44) that often encourage boys to engage in competitive, team-based sports from an early age. As noted by Andersson (28), the training structures and curricular emphasis in many European sport schools reflect historical trends that have shaped gendered participation patterns, potentially privileging boys' involvement in certain sports while limiting comparable opportunities for girls. For example, these practices may increase boys' exposure to motor tasks such as throwing and kicking, whereas girls often encounter fewer chances to develop these skills (44, 45). Conversely, female students demonstrated relatively stronger performance in coordination-oriented tasks like jumping rope and forward roll. This may partly reflect earlier neuromuscular maturation (46) as well as anthropometric characteristics, such as shorter stature and a lower center of mass, that could facilitate balance and coordination (47). Additionally, cultural preferences that promote aesthetic movement forms more commonly associated with activities like gymnastics or dance may further support performance in these skill domains (43). These findings underline the importance of providing all students with opportunities to practice a wider variety of motor tasks, regardless of sex.

Building on this, our data also revealed that patterns of proficiency varied not only by sex but also by sport specialization. For example, girls in track and field demonstrated strong performance in hurdles and forward rolls, whereas volleyball participants excelled in throwing and catching. Basketball participants demonstrated the highest proficiency in ball bouncing and jumping rope, but their performance in hurdles was comparatively lower than their peers in track and field. Similarly, soccer boys excelled in kicking and stopping a ball, yet they showed weaknesses in coordination-oriented tasks such as forward roll and jumping rope. Volleyball participants of both sexes displayed moderate proficiency across most tasks, without leading in any specific domain. These findings are consistent with recent evidence highlighting how different sport environments – especially open- vs. closed-skill disciplines – shape distinct motor competence profiles in youth athletes (48, 49). For instance, gymnastics (a closed-skill sport) often fosters higher balance and coordination abilities due to its technical demands, whereas team sports like soccer emphasize object-control skills and reactive motor behaviors under dynamic conditions (48). Similarly, Spanou et al. (49) reported that children's motor competence varies considerably by sport type, even when cognitive functions remain comparable across disciplines. These insights reinforce the need for PE programs that not only focus on sport-specific skills but also promote balanced motor development across various domains to avoid narrow proficiency profiles and potential long-term limitations.

This study provides valuable insights, though certain limitations must be acknowledged. The sample was drawn from students participating in a specific nationwide sport education program in Poland, which may limit the applicability of the findings to other educational or cultural contexts. In addition, individual physical characteristics such as stature, body mass, and biological maturation were not measured, even though these factors are known to influence motor performance, particularly in tasks where anthropometric attributes are relevant. Although the FUS test has demonstrated strong psychometric properties, including excellent inter- and intra-rater reliability and validity, no assessment tool is without limitations. Ceiling effects were not observed in this study, but the potential for contextual biases and floor effects in populations with very low FMS proficiency warrants further investigation. The cross-sectional nature of the study further restricts the ability to track developmental changes over time. To address these gaps, future research should employ longitudinal designs to explore how different instructional approaches, particularly those that emphasize early specialization or diversification, shape FMS proficiency, athletic potential, and long-term PA behaviors.

The present findings indicate that while students enrolled in school-based, sport-focused PE programs demonstrated higher overall FMS proficiency compared to their peers in traditional settings, the majority still failed to meet even “elementary” proficiency benchmarks. This proficiency profile suggests that such programs often emphasize sport-specific training – potentially at the expense of broader FMS development – and exhibit characteristics commonly associated with early specialization. Such programming may increase the risk of burnout, injury, and dropout, ultimately hindering both talent development and lifelong PA engagement. These results should not be seen as merely descriptive but rather as diagnostic, exposing structural limitations within current sport-oriented PE models. They suggest that, despite well-intentioned efforts to nurture talent, many existing pathways may inadvertently compromise long-term athletic development by neglecting the FMS proficiency necessary to support diverse sport goals.

## Data Availability

The original contributions presented in the study are included in the article/Supplementary Material, further inquiries can be directed to the corresponding author.
